# Comprehensive analysis of the 5-HTTLPR allelic polymorphism effect on behavioral and neurophysiological indicators of executive control in people from different ethnic groups in Siberia

**DOI:** 10.18699/VJ21.066

**Published:** 2021-09

**Authors:** A.N. Savostyanov, D.V. Bazovkina, S.А. Lashin, S.S. Tamozhnikov, A.E. Saprygin, T.N. Astakhova, U.N. Kavai-ool, N.V. Borisova, A.G. Karpova

**Affiliations:** Institute of Cytology and Genetics of the Siberian Branch of the Russian Academy of Sciences, Novosibirsk, Russia Scientific-Research Institute of Neuroscience and Medicine, Novosibirsk, Russia Novosibirsk State University, Novosibirsk, Russia; Institute of Cytology and Genetics of the Siberian Branch of the Russian Academy of Sciences, Novosibirsk, Russia; Institute of Cytology and Genetics of the Siberian Branch of the Russian Academy of Sciences, Novosibirsk, Russia Novosibirsk State University, Novosibirsk, Russia; Scientific-Research Institute of Neuroscience and Medicine, Novosibirsk, Russia; Scientific-Research Institute of Neuroscience and Medicine, Novosibirsk, Russia; Novosibirsk State University, Novosibirsk, Russia; Tuvan Scientific Center, Kyzyl, Tyva Republic, Russia; M.K. Ammosov North-Eastern Federal University, Yakutsk, Sakha Republic, Russia; M.K. Ammosov North-Eastern Federal University, Yakutsk, Sakha Republic, Russia

**Keywords:** serotonin transporter, 5-HTTLPR polymorphism, personal anxiety, stop-signal paradigm, premotor evoked potential, транспортер серотонина, полиморфизм 5-HTTLPR, личностная тревожность, парадигма стопсигнал, премоторный вызванный потенциал

## Abstract

The allelic polymorphism of the serotonin transporter's gene 5-HTTLPR is considered as one of the factors determining an individual genetic predisposition to the development of a wide range of affective disorders, including depression. Many studies have shown that the climatic and social conditions of people's life can have a significant impact on the connections of 5-HTTLPR with the risk of depression. The stop-signal paradigm (SSP) is an experimental method allowing evaluating an individual ability to the self-control of behavior in a changing environment. In the SSP experiment, a subject should either press one of several buttons quickly after the appearance of the target stimuli or suppress the already started movement if an inhibitory signal follows the target stimulus. The aim of this study is a research of associations between the allelic the 5-HTTLPR polymorphism and the individual scores of the personal anxiety level, as well as the behavioral and neurophysiological indicators of the ability to self-control over motor reactions in the SSP. The study was conducted among people from three ethno-regional groups: healthy Caucasoids from Novosibirsk, the Mongoloid groups of the indigenous population of the Tuva Republic and Sakha Republic (Yakutia). Genetic, ethnographic, and psychological influences on an individual's ability to control motor responses were compared. The amplitude of the premotor peak of the evoked brain potential was used as a neurophysiological marker of the person's readiness to the execution of target-directed activity. It was revealed that the frequency of the S-allele polymorphism 5-HTTLPR was significantly higher for both mongoloid groups compared to the Caucasoids. The S/S genotype was associated with an increased level of personal anxiety and at the same time with a better ability to the self-control of behavior in the SSP experiment. Anxiety level, participants' sex, ethnicity, and allelic polymorphism 5-HTTLPR had a statistically significant effect on the amplitude of the premotor readiness potential recorded under the SSP conditions in the frontal and parietal-occipital cortical regions. Our data support the hypothesis that the S/S genotype of the 5-HTTLPR polymorphism may be associated with more success in adapting to the climatic conditions connected with high life risk in comparison to L/L and L/S genotypes.

## Abstract (ru)

Аллельный полиморфизм гена транспортера серотонина 5-HTTLPR рассматривается как один из факторов, определяющих генетическую предрасположенность человека к развитию широкого ряда аффектив- ных нарушений, включая депрессию. Во многих исследованиях было показано, что климатические и социаль- ные условия жизни людей могут оказать существенное влияние на взаимосвязь 5-HTTLPR с риском депрессии. Стоп-сигнал парадигма (ССП) – это экспериментальный метод, позволяющий оценить способности человека к контролю своего поведения в условиях изменяющейся внешней среды. В ССП эксперименте испытуемый дол- жен либо быстро нажимать на одну из нескольких кнопок после появления целевых стимулов, либо подавлять уже начатое движение, если после целевого стимула следует запрещающий сигнал. В представленной работе исследуются ассоциации полиморфизма 5-HTTLPR с оценками уровня личностной тревожности, а также пове- денческими и нейрофизиологическими (электроэнцефалограмма, ЭЭГ) показателями способности к контролю двигательных реакций в ССП у представителей трех этно-региональных групп – здоровых европеоидов из г. Но- восибирска, монголоидных групп коренного населения Республики Тыва и Республики Саха (Якутия). Целью ис- следования было сопоставление генетических, этнографических и психологических эффектов на способность индивида к контролю моторных ответов. Амплитуда премоторного пика вызванного потенциала использована в качестве нейрофизиологического маркера готовности человека к выполнению целенаправленных действий. Выявлено, что частота встречаемости аллеля S полиморфизма 5-HTTLPR достоверно выше для обеих монго- лоидных групп в сравнении с европеоидной. Генотип S/S ассоциирован с повышенным уровнем личностной тревожности и одновременно с лучшей способностью к контролю движений в условиях ССП эксперимента. Уровень тревожности, пол испытуемых, этническая принадлежность и аллельный полиморфизм 5-HTTLPR ока- зывают статистически достоверное влияние на амплитуду премоторного потенциала готовности, регистрируе- мого в условиях ССП в лобных и теменно-затылочных областях коры. Наши данные подтверждают гипотезу, что генотип S/S полиморфизма 5-HTTLPR может быть ассоциирован с большей успешностью адаптации к климати- ческим условиям, связанным с высоким риском для жизни, в сравнении с генотипами L/L и L/S.

## Introduction

The subject of psychological genetics is the identification of molecular markers associated with psychological characteristics of healthy people and predisposition to the onset of psychiatric and neurological diseases (Eysenck, 1990; Miller, Lynam, 2003). Serotonin neurotransmitter transporter (5-HTT) polymorphism is one of the most extensively studied molecular markers of predisposition to a wide range of mental disorders (Lesch et al., 1997; Arango et al., 2003). In humans, the serotonin transporter is encoded by the SLC6A4 gene located on chromosome 17 (Gelernter et al., 1995). The promoter region of the serotonin transporter gene (5-HTTLPR) contains 16 tandem repeats about 20 bp units. Polymorphism 5-HTTLPR is presented by two allelic variants: long variant contains 16 repeats (L allele) and short variant contains 14 repeats (S allele). It is known, the S allele is associated with a reduced efficiency of the transport function of this protein (Lesch et al., 1996). In addition, the long allele contains an A/G single nucleotide polymorphism, with the Lg allele functionally similar to the S allele (Hu et al., 2005).

It was revealed, that the S allele increased the risk of depression in people who have experienced life stress (Caspi et al., 2003). However, the relationship of this allele with stress and depression is still a topic of active discussion (Munafò et al., 2009; Risch et al., 2009; Knyazev et al., 2017). In some works, this connection was confirmed, while in others, on the contrary, it was rejected. There are also studies in which the S allele is associated not only with negative qualities. Anumber of studies have shown that people with the S allele performed better than carriers of the L/L genotype when solving a wide range of cognitive tasks (Homberg, Lesch, 2011). S allele carriers showed greater success on the divergent thoughts test (Volf et al., 2009), demonstrated better visual planning abilities (Roiser et al., 2006) and better attention to differences in the probability of winning, showed more intensive error handling (Althaus et al., 2009), high performance in card sorting tests in the Wisconsin problem (Borg et al., 2009), and higher IQ scores (Volf et al., 2015). All of these results can be explained by the “differential susceptibility” hypothesis, according to which the so-called “risk alleles” may have a higher sensitivity to environmental challenges. What is harmful in some circumstances may be beneficial in other life situations.

The association of 5-HTTLPR polymorphism with personality traits in healthy individuals is also a topic of intense debates (Hariri et al., 2005; Dannlowski et al., 2008; van der Meer et al., 2016). We have previously shown that associations between different 5-HTTLPR alleles and personality traits in healthy subjects were significantly modulated by ethnic and cultural affiliation of people (Savostyanov et al., 2015). In general, from a review of the scientific literature, we can conclude that when searching for markers of mental illness or personality traits, it is impossible to limit ourselves only to the molecular-genetic level of the description of the nervous system. It is also necessary to take into account the characteristics of human behavior in changing environmental conditions, including their behavior in society.

When studying complex multifactorial associations between genotype and behavior, it is paramount to choose a proper experimental model that will allow to conduct the study. One of these models is the stop-signal paradigm (SSP, Band et al., 2003). SSP is designed as a method to assess a person's ability to control their own actions in a changing environment with an acute shortage of time for decision-making. The essence of the method is that a person in a random order either performs quick targeted actions in response to the appearance of target events, or suppresses the already started activity if the target event is followed by a prohibitory signal. Previously we showed that 5-HTTLPR polymorphism is associated with indicators of motor control under ERP conditions (Karpova et al., 2017). Carriers of the S allele showed significantly better scores on control over movements compared to people with the L/L genotype. In another our study, it was shown that SSP can be used to study endophenotypic differences between subjects (Savostyanov et al., 2009). Comparison of EEG reactions under ERP conditions revealed significant differences between people with different levels of anxiety regarding the dynamics of neurophysiological processes associated with control over movements. Thus, on the basis of preliminary studies, it can be concluded that the analysis of behavioral and neurophysiological indicators recorded in the SSP experiments makes it possible to reveal their dependence simultaneously on genetic differences of people, the level of their personal anxiety and other indicators including gender and age of participants.

The current article presents the multivariate analysis of the associations between the 5-HTTLPR allelic polymorphism, the level of personal anxiety, and behavioral and neurophysiological indicators reflecting brain activity under motor control conditions. One of the well-studied neurophysiological markers of motor control detected in ERP is the premotor readiness potential (the so-called Bereitschaftspotential or readiness potential). This electrographic brain response is detected on the EEG in time intervals immediately preceding the execution of the planned actions. The amplitude and cortical topography of the premotor potential reflects a person's readiness to perform an action.

We carried out a comparative study in three groups of healthy young people, differing in ethnicity and region of residence. Alarge group of Caucasians (mainly Russians), permanently residing in a large industrial city (Novosibirsk), was examined. In addition, two independent groups of Siberian Mongoloids were examined – Tuvans living in the Republic of Tuva, Kyzyl, and a group of Mongoloids, consisting mainly of Yakuts and Evenks, living in the Republic of Sakha (Yakutia). Our hypothesis assumed that the influence of the level of anxiety and 5-HTTLPR polymorphism affects the amplitude of the cerebral premotor potential under conditions of movement activation in the ERP.

## Materials and methods

Subjects. A total of 294 young, healthy subjects, in average 23.4±3.2 y. o., 117 men, 177 women, mainly students of various universities participated in the survey. 121 of them were permanently living in Novosibirsk and considered themselves to be one of the Caucasian ethnic groups (approximately 72%– Russians, 10%– Ukrainians, 7%– Tatars, 4%– Jews, 7 % – the rest). 94 people were examined at the Tuva State University, Kyzyl, the Republic of Tuva. All subjects from this group referred to themselves as ethnic Tuvans. Another 79 people were examined at the North-Eastern Federal University in Yakutsk. In this group, approximately 80 % identified themselves as Yakuts (Sakha), 15 % – as Evenks and about 5 % – as Yukagirs.

Before the examination, all subjects signed an informed consent to participate in the examination and filled out a questionnaire in which they noted the presence of various diseases. The exclusion criteria were the presence of mental or neurophysiological diseases, as well as brain injuries within three years before the examination. In addition, participants who indicated in the questionnaire that they use drugs or psychoactive substances were excluded from the experimental sample. Also, pregnant women and women in the first four days of the menstrual cycle were excluded from the study. The examination protocol met the requirements of the Declaration of Helsinki on Biomedical Ethics and was approved by the Ethics Committee of the Research Institute of Physiology and Fundamental Medicine.

Before the start of the examination, all subjects completed the Russian version of the psychological questionnaire of C. Spielberger to assess the level of situational and personal anxiety. In addition, buccal epithelium samples were taken from all participants to determine the 5-HTTLPR allelic polymorphism.

Determination of 5-HTTLPR polymorphism. Genomic DNA was isolated from buccal epithelium samples by using a DNA isolation kit (Biosilica, Russia). The genotypes of the subjects (L/L, L/S, S/S) were determined by PCR using specific primers F 5'-ggcgtgcgctgtgaattgc-3' and R 5'-gagga ctgagctgacaaccac-3' (Lesch et., 1996) and the genomic DNA of the subjects as a template. PCR products were separated by electrophoresis in 3 % agarose gel stained with ethidium bromide. The S and L allele sizes for 5-HTTLPR were 489 and 529 bp, respectively. For determination of polymorphism of La/Lg the products of amplification split during 3 h by MspI endonuclease. The resulting fragments were separated and visualized on 3 % agarose gel stained with ethidium bromide. Cleavage product sizes for the La allele were 340, 127 and 62 bp, while for the Lg allele were 174, 166, 127 and 62 bp. Lg allele was included in the S allele group because the two alleles are functionally similar (Hu et al., 2005).

Experimental method “Stop-Signal Paradigm”. In the experiment, the technique proposed by Band et al. (2003) and adapted for EEG recording by Savostyanov and co-authors (2009) was used. The experiment was designed in the form of a computer game “Hunt”. The participant was randomly presented with 130 visual stimuli (50% tanks and 50% deers). Stimuli with a size of 5×7 cm appeared in the center of the computer screen, located about 1 meter from the subject's head. The stimulus was shown on the screen for 0.75 seconds; the interval between stimuli varied randomly within 3–5 seconds. The subject's task was to press the left button as quickly as possible after the appearance of a deer (which corresponded to a shot from a crossbow) and the right button after the appearance of a tank (which corresponded to a shot from an anti-tank weapon). If the participant managed to press the button correctly before the image disappeared from the screen, they were awarded game points. If the participant chose the button incorrectly or pressed it after 0.75 seconds, their game score was decreased. In 35 % of cases, after the appearance of the target stimulus, a stop signal was presented (a red square in the center of the figure with the inscription “Stop”). The interval between the appearance of the target light and the stop light ranged from 0.25 to 0.75 seconds. In the event of a stop light, the participant had to interrupt the movement that had already started. If the participant stopped moving, their score did not change. If the subject pressed the button after the stop-light appeared, their score would decrease. Accordingly, all tasks of the SSP were subdivided into the “Go” condition, when it was necessary to press the button (100 tasks out of 130), and the “Stop” condition (30 tasks out of 100), when it was necessary to suppress the movement. The sequence of tasks from both conditions was randomized for all subjects.

EEG registration. EEG was recorded using an actiChamp biopotential amplifier from Brain Products, Germany, with a bandwidth of 0.3–100 Hz, a signal sampling rate of 1000 Hz. The electrodes were placed according to the international scheme 10–5 % with grounding at AFz and reference at Cz. For participants from Novosibirsk, EEG was recorded using 128 channels, for participants from Kyzyl and Yakutsk, EEG was recorded using 64 channels. Additionally, an electrooculogram (VEOG, HEOG) and an ECG were recorded for all participants

EEG preprocessing and calculation of event-related potentials. To assess changes in signal amplitude associated with the appearance of a target stimulus, event-related potentials (ERPs) were calculated using the ERPLAB software package ( https://erpinfo.org/erplab ). EEG fragments containing muscle artifacts that could not be corrected were excluded from the analysis. For each participant, 80–90 EEG fragments containing the mark of the target event occurrence in the “Go” condition were selected. The time interval from –1.5 to +3.0 seconds before and after the appearance of the target signal was selected for analysis. The time interval from –1.5 to –0.5 seconds before the appearance of the target signal was used for baseline correction.

The EEG was preliminarily f iltered in the range of 1–40 Hz using eleptic f ilters. Following the recommendations of Delorme and Makeig (2004), the re-reference procedures for the averaged referent and subtraction of the baseline were performed during data pre-processing. Independent components analysis (ICA) was performed to exclude oculomotor and blink artifacts. Initially, component weights were calculated individually for each participant. Components that corresponded to ocular artifacts were identified by visual inspection in conjunction with EOG and ECG. Components with artifacts were removed during EEG pre-processing.

Event-related potentials (ERPs), which were calculated in the ERPLAB software package, were used to assess changes in brain activity associated with motor tasks. After removing the artifacts, we calculated the ERP values using the ERPLAB program independently for each EEG channel and each participant. The results were f iltered with a 15 Hz cutoff f ilter. After that, the average amplitude for the premotor readiness peak was calculated in a time window from 350 to 600 ms after the appearance of the target stimulus.

Statistical analysis of results. One Way analysis of variance (ANOVA) with one target and three fixed variables was used to statistically assess the significance of the obtained behavioral results. As a target variable, one of three indicators was chosen independently of each other – the level of personal anxiety, time (in milliseconds) and quality (percentage of correct decisions) of the task in the “Go” condition. The fixed variables were simultaneously stated: “gender” (men or women), “ethno-regional group” (Novosibirsk Caucasians, Tuvinians, Yakut groups), “5-HTTLPR polymorphism” (L/L, L/S or S/S genotypes).

To process the amplitude of the event-related potentials, the data obtained for each EEG channel were averaged over 11 groups of electrodes corresponding to the left frontal, medial frontal, right frontal, left temporal, right temporal, left central, medial central, right central, left parieto-occipital, medial parieto-occipital and the right parieto-occipital regions of the cerebral cortex. After that, multivariate analysis of variance ANOVA was applied with repeated measurements and Greenhouse–Geisser sphericity correction with the factors “cortical regions” (11 sections of the cortex), “sex” (men or women), “ethno-regional group” (Novosibirsk Caucasians, Tuvinians, Yakut group), “5-HTTLPR polymorphism” (L/L, L/S or S/S genotypes), “anxiety level” (people with relatively low or relatively high anxiety, the sample was divided according to the median value of anxiety scores).

## Results

Prevalence of 5-HTTLPR alleles in regional groups

The prevalence of various alleles of 5-HTTLPR polymorphism among subjects from the cities of Novosibirsk, Kyzyl and Yakutsk is presented in the Table. Among the representatives of the Caucasian sample from Novosibirsk, the frequency of occurrence of the L allele (56.6 %) exceeded the frequency of the S allele (43.4 %), while in both Mongoloid samples, on the contrary, the S allele was found significantly more often than the L allele. When comparing the three groups, there were four degrees of freedom, the boundary value of χ^2^ for three degrees of freedom is more than 9.50; between the examined groups χ^2^ = 23.55, the significance of intergroup differences when comparing the Novosibirsk Caucasoid and two Mongoloid samples was p < 0.01 (see the Table).

**Table.**
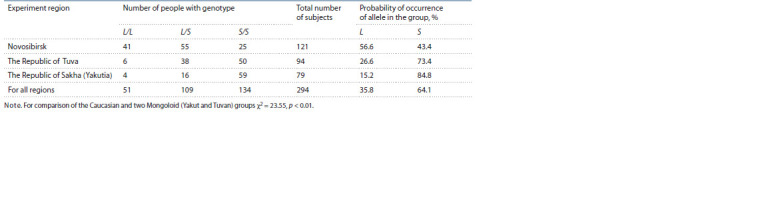
Frequency of occurrence of L and S alleles of 5-HTTPLR polymorphism in the regional groups of the subjects Notе. For comparison of the Caucasian and two Mongoloid (Yakut and Tuvan) groups χ^2^ = 23.55, p < 0.01.

It was important to note that in the sample of Yakuts and Evenks, the frequency of occurrence of the S allele (84.8 %) was higher than in the sample of Tuvans (73.4 %). When comparing the Yakut and Tuvan groups, there were two degrees of freedom, the boundary value of χ^2^ for two degrees of freedom was more than 5.99; between the two examined groups χ^2^ = 8.30, the significance of intergroup differences when comparing two Mongoloid (i.e. Yakut and Tuvan) samples among themselves was p <0.03. Thus, the two Mongoloid samples differed from each other in the prevalence of alleles of the 5-HTTLPR polymorphism, although not as contrastingly as they both differed from the Caucasian sample.

Association of effects of group, gender and 5-HTTLPR polymorphism with the level of personal anxiety

**Fig. 1.**
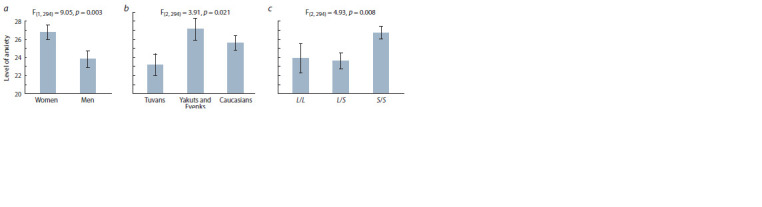
Relationship between the level of personal anxiety and gender (a), ethnic and regional aff iliation of the participants (b), and 5-HTTLPR polymorphism (c).

The level of personal anxiety assessed using the Spielberger questionnaire significantly differed between participants of different sex. Anxiety was significantly higher for women (mean 26.8±0.8) than for men (mean 23.8±0.9), F_(1, 294)_= 9.05; p = 0.003; η^2^ = 0.050 (Fig. 1, a). Also, the main effect of the “group” factor was revealed when comparing the assessments of anxiety for representatives of the subjects from different regions, F_(2, 294)_ = 3.91; p = 0.021; η^2^ = 0.053 (see Fig. 1, b). The minimum anxiety was found in the Tuvin group (mean 23.2±1.2), and the maximum in the Yakut group (mean 27.1±1.2), while in the Caucasian group, the average level of anxiety was intermediate between the two Mongoloid groups (25.6±0.8).

The main effect of 5-HTTLPR polymorphism on the level of personal anxiety was statistically significant, F_(2, 294)_= 4.93; p = 0.008; η^2^ = 0.032 (see Fig. 1, c). The level of anxiety for carriers of genotypes L/L (mean 23.9 ± 1.6) and L/S (23.6 ± 0.9) was significantly lower than for people with the genotype S/S (26.7±0.7). Post-hoc comparisons did not reveal significant differences in the level of anxiety between people with the L/L and L/S genotypes (p > 0.5), but revealed a significant difference between people with the S/S genotype and carriers of two other genotypes (p < 0.03).

The factors of gender, group and 5-HTTLPR polymorphism on the level of anxiety did not significantly interact with each other. Calculation of the effect of each of these factors under the control of other factors did not lead to the disappearance of the significance of the effects, although the significance of the 5-HTTLPR factor in this case slightly decreased ( p = 0.008 excluding group and sex vs p = 0.035 under the control of group and sex). Thus, the influence of these three factors on the level of anxiety can be considered as independent of each other.

Association of the effects of group, gender, personality anxiety, and 5-HTTLPR polymorphism with behavioral indicators in the stop-signal parad

For indicators assessing a person's ability to suppress movements after stop-signal onset, no significant effects of gender, group or genotype, as well as their interactions, were identified.

A significant effect of the group was revealed for the reaction time in the “Go” condition, F_(2, 276)_ = 3.66; p = 0.052; η^2^ = 0.013. Tuvans (mean time 561 ± 3 ms) and Yakuts (562±4 ms) showed faster reaction time in comparison with Caucasians (569 ± 3 ms). Post-hoc comparisons did not reveal significant differences in reaction time between the Tuvan and Yakut groups ( p > 0.7), but revealed differences between Caucasians from both other groups (p < 0.05). Also, for the response time, a significant effect of gender was revealed, F_(1, 276)_ = 3.72; p = 0.055; η^2^ = 0.013. The average reaction time was lower for men (561±3 ms) than for women (568±3 ms). The effect of 5-HTTLPR polymorphism or its interaction with other effects for the response time was statistically not significant

**Fig. 2.**
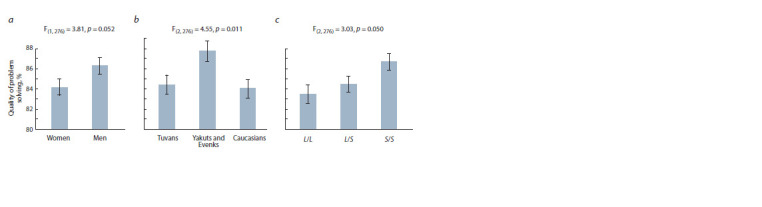
The relationship between the quality of problem solving in the “Go” condition of the stop-signal paradigm with gender (a), ethnic and regional aff iliation of participants (b), and 5-HTTLPR polymorphism (c).

For the indicator of the quality of performance of tasks in the condition “Go”, a significant main effect of the factor “gender” was revealed, F_(1, 276)_ = 3.81; p = 0.052; η^2^ = 0.014 (Fig. 2, a). Men performed this task with better average quality (86.3±0.8 %) than women (84.2±0.8 %). Also, for the quality indicator, the main effect of the “group” factor was significant, F_(2, 276)_ = 4.55; p = 0.011; η^2^ = 0.032 (see Fig. 2, b). Tuvans (84.3±0.9 %) and Caucasians (84.0±0.9 %) showed the same average quality of task performance, while the average quality of task performance in the Yakut group (87.7±1.0 %) was significantly higher than in both other groups. The main effect of 5-HTTLPR polymorphism, calculated without control for other factors, was statistically marginal (p = 0.090). However, when calculating the effect of polymorphism under the control of the level of personal anxiety, it became significant, F_(2, 276)_ = 3.03; p = 0.050; η^2^ = 0.019 (see Fig. 2, c). People with the S/S genotype showed the best average quality of solving motor tasks (86.7±0.8 %), in comparison with carriersof genotypes L/S (84.5±0.8 %) and L/L (83.5±0.9 %). The effect of the level of anxiety on indicators of speed or quality of solving motor tasks was not revealed. The factors of gender, group and polymorphism of the serotonin transporter did not significantly interact with each other 

In general, based on the results of the analysis of the effects of factors of gender, regional-ethnic group and 5-HTTLPR polymorphism on psychological and behavioral indicators, it can be concluded that all three selected factors affect both the level of anxiety and the indicators of motor control. However, their effects did not significantly affect each other. It can also be noted that, although 5-HTTLPR simultaneously affected both the level of anxiety and the quality of motor control, none of the indicators of motor control were directly dependent on the level of personal anxiety.

Association of the effects of group, gender, personality anxiety and 5-HTTLPR polymorphism with the amplitude of the premotor event-related potentials in the stop-signal paradigm.

**Fig. 3.**
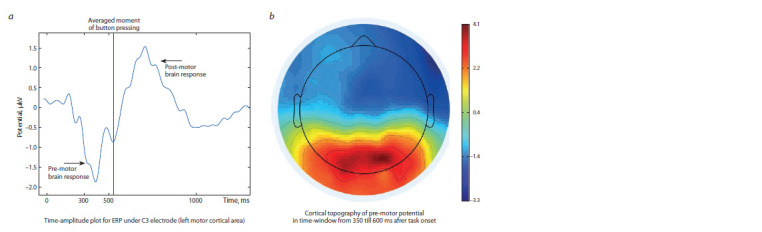
Amplitude-time graph (a) and cortical topography (b) for cerebral ERP responses in the “Go” condition of SSP.

The amplitude-time graph of the event-related potentials in the left motor area and the topographic amplitude distribution of the premotor potential are shown in Fig. 3. Initially, the influence of various factors on the amplitude of the premotor event-related potentials was simultaneously assessed for all 11 cortical regions. With this method of assessment, only significant effects of the region were revealed, F_(10, 2920)_ = 300.05; p < 0.0001, and group F_(2, 294)_ = 4.30; p = 0.014. The premotor potential had a negative amplitude in the frontal and temporal regions of the cortex and a positive amplitude in the central and parieto-occipital regions. The amplitude in the left (r = –0.18; p = 0.003) and right (r = –0.15; p = 0.011) frontal lobes negatively correlated with the quality of task solution under “Go” conditions, and in the medial central (r = 0.17; p = 0.005) and the medial parieto-occipital (r = 0.14; p = 0.024) areas, these correlations were positive. If we take into account that the selected peak had a negative polarity for the frontal areas and positive for the central and parieto-occipital areas, we can conclude that its large amplitude in magnitude corresponded to the best quality of tasks in all areas of the cortex.

Interactions of factors “region” to “group”, F_(20, 2940)_ = = 21.28; p < 0.0001; η^2^ = 0.195, “region” to “5-HTTLPR polymorphism”, F_(20, 2940)_ = 5.81; p < 0.0001; η^2^ = 0.046, “region” to “level of anxiety”, F_(10, 2950)_ = 2.38; p = 0.008; η^2^ = 0.049, and “region” to “sex”, F(10, 2920) = 3.99; p = 0.011; η^2^ = 0.061 were statistically highly significant. The effects of all factors appeared only in the frontal and occipital-parietal regions of the cortex and did not affect other regions. In addition, since the directivity of the peak amplitude in the anterior (negative) and posterior (positive) regions was different, the statistical analysis was performed separately for the frontal and occipital-parietal regions.

In the frontal parts of the cortex, a significant main effect of the group was revealed, F_(2, 294)_ = 8.91; p < 0.0001; η^2^ = 0.023. The negative peak amplitude was the maximum modulus in the Yakut group (–2.0 ± 0.2 μkV), less in the maximum modulus in the Tuvan group (–1.7±0.1 μkV) and the lowest in the maximum modulus in the Caucasian group (–1.2± 0.1 μkV). Post-hoc comparisons revealed pairwise significant differences in the frontal negative amplitude of the premotor peak between all three groups (p <0.01). The effect of the gender factor for this indicator was also significant, F_(1, 292)_ = 8.30; p = 0.004; η^2^ = 0.030. The negative amplitude was higher in absolute value for women (–1.8 ±0.1 μkV) than for men (–1.3 ± 0.1 μkV). The main effect of 5-HTTLPR polymorphism was significant, F_(2, 294)_ = 3.90; p = 0.021; η^2^ = 0.026. The amplitude of the negative peak was greater in modulus for carriers of the S/S genotype (–1.8±0.1 μkV) than for people with the L/S (–1.4 ± 0.1 μkV) and L/L (–1.3 ± 0.2 μkV) genotypes. No significant interactions were found for all selected factors. The effect of personality anxiety and its interaction with other effects in the frontal cortex was also not significant.

**Fig. 4.**
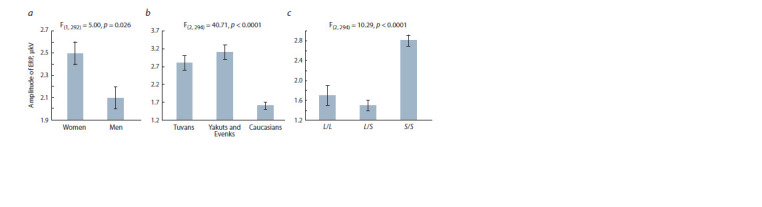
Correlation of the amplitude of the premotor peak of the event related potential in the “Go” condition of ERP in the parietooccipital cortex with sex (a), ethno-regional affiliation of participants (b), and 5-HTTLPR polymorphism (c).

In the parieto-occipital parts of the cortex, significant effects of sex were revealed, F_(1, 292)_ = 5.00; p = 0.026; η^2^ = 0.033, group, F_(2, 294)_= 40.71; p < 0.0001; η^2^ = 0.218, and 5-HTTLPR polymorphism, F_(2, 294)_= 10.29; p <0.0001; η^2^ = 0.065 (Fig. 4). The positive amplitude in the posterior parts of the cortex was maximum in the Yakut group (3.1±0.2 μkV), less in the Tuvan group (2.8±0.2 μkV) and the lowest in the Caucasian group (1.6±0.1 μkV). Post-hoc comparisons revealed pairwise significant differences in the parieto-occipital amplitude of the premotor peak between all three groups (p < 0.01). The positive amplitude was higher for women (2.5±0.1 μkV) than for men (2.1±0.1 μkV). The amplitude of the positive peak was greater for carriers of the S/S genotype (2.8 ± 0.1 μkV) than for people with genotypes L/S (1.5 ± 0.1 μkV) and L/L (1.7 ± 0.2 μkV). As for the frontal cortex, in the parieto-occipital cortex, no significant interactions were found for all selected factors.

When calculating the effect of personal anxiety simultaneously for three areas of the parieto-occipital cortex, this effect was insignificant. However, it turned out to be significant separately for the left (F_(1, 296)_ = 3.93; p = 0.048; η^2^ = 0.013, the amplitude for low-anxious people was lower (1.1±0.1 μkV) than for highly anxious (2.5 ±0.2 μkV)) and for the right one (F_(1, 296)_ = 6.19; p = 0.013; η^2^ = 0.021, the amplitude for low-anxious people was lower (1.9±0.2 μkV) than for highly anxious (2.5±0.2 μkV)) parieto-occipital areas and was not significant for the medial parieto-occipital cortex ( p > 0.5).

Thus, based on the analysis of the amplitude of the premotor event-related potential, the effects of group (the strongest brain responses in Yakuts, the weakest in Caucasians), gender (the amplitude of responses was greater in women than in men), and 5-HTTLPR polymorphism (the highest responses in carriers of the S/S genotype) and anxiety (stronger in highthan in low-anxious) were identified. The amplitude of the premotor potentials correlated with the behavioral indicator of the quality of task solving. However, the effects for all the factors we selected on the premotor potential amplitude did not interact with each other.

Discussion

The frequency of occurrence of S and L alleles of 5-HTTLPR polymorphism in Caucasoid and Mongoloid samples revealed is consistent with well-known patterns obtained by comparing different ethnic groups (see Esau et al., 2008; Noskova et al., 2008; Ivanov et al., 2019). It is well known that the L allele is more common in Caucasians, while the S allele is more common in Mongoloids. Our data are broadly consistent with these results. It can be noted that Caucasians from Novosibirsk are more likely to carry the S allele than Caucasians from Europe, the United States and even from the European part of Russia. Among Siberian Mongoloids, the frequency of occurrence of this allele is higher in Yakuts and Evenks in comparison with Tuvans. This indirectly indicates the relationship of the S allele with increased adaptability to extreme or sub-extreme climate conditions. Indeed, in the series Western Europe – Western Siberia – Southeast Siberia and Northeast Siberia, extreme climatic conditions for human life are escalating. The frequency of the S allele also increases from West to North-East. Although at present we do not have direct data indicating a relationship between 5-HTTLPR polymorphism and mechanisms of adaptation to extreme climates, we can assume the existence of such an association as a working hypothesis.

This hypothesis is supported by the relationship of 5-HTTLPR with behavioral indicators of motor control and the level of anxiety. In the modern psychological literature, anxiety is usually viewed as a negative marker associated with an increased risk of a number of diseases, such as depression, or psychosomatic disorders. However, in conditions accompanied by an increased danger to life, anxiety should serve as an adaptive factor that reduces the risk of human death. It can be noted that the genetic marker of high anxiety (S allele) is most common in groups of people living in subpolar or polar climates. The same allele is a marker associated with higher rates of motor control in an experimental model assessing the ability to self-regulate behavior under time pressure. The facts above allowed to formulate the assumption that the S allele, which is “bad” from the point of view of the urban environment, may turn out to be a marker of increased ability to adapt in conditions associated with high danger to life.

As mentioned above, the S allele is associated with a reduced efficiency of the transport function of this protein. Biochemical studies showed that animals with the S/S genotype were characterized by a reduced level of serotonin in the sy-naptic cleft and a reduced level of functional activity of serotonergic neurons (Lesch et al., 1996). It is also known that the serotonergic system in the regulation of behavior is responsible for the performance of inhibitory control (Munafò et al., 2009). According to literature data obtained in psychiatric patients, it is known that the S/S genotype should be associated with the lower ability to delay irrelevant behavioral responses (Malloy-Diniz et al., 2011). However, our data under the “Stop” condition in the SSP did not reveal differences between carriers of different 5-HTTLPR alleles in either behavioral or ERP parameters. It can be assumed that in healthy people with the S/S genotype, a decrease in the activity of 5-OHT neurons is associated not with a deterioration in inhibitory control, but with an improvement in the parameters of activation control due to a lower suppression of motor neurons. In this case, a decrease in the concentration of serotonin in the brain due to a decrease in the transport function of the carrier protein under some external conditions can be considered as a marker of an increased tendency to impulsive-anxious behavior, and under other living conditions – as a mechanism of adaptation to high danger

It can also be noted that, in addition to the allelic polymorphism chosen, the behavioral indicators of motor control and the level of anxiety are influenced by several other factors independent of each other. Women are, on average, more anxious than men. Caucasians are more anxious than Tuvans, but less anxious in comparison with Yakuts and Evenks. Men are better at motor control tasks than women. Mongoloids perform these tasks on average faster and better than Caucasians. At the same time, no statistical interaction of the factors we selected was found. The occurrence of the S allele associated with high anxiety was higher in both Mongoloid groups in comparison with the Caucasoids, but at the same time, Tuvinians are less anxious, and Yakuts and Evenks are more anxious than Caucasians. Thus, both the level of anxiety and the ability to control movements are determined not by one, but by a wide range of factors which interact unclearly.

The attempt made in the framework of this study to find a mechanism for integrating the effects of genetic and environmental factors using the analysis of cerebral event-related potentials has not yet given a completely satisfactory result. We have confirmed the previously established fact that the frontal and parieto-occipital amplitude of the premotor readiness potential correlates with the success of solving motor tasks. We have also shown that the amplitude of this potential depends on the 5-HTTLPR allelic polymorphism. People with the S/S genotype show both increased abilities for movement control of behavior and an increased amplitude of premotor cerebral responses to EEG in the frontal and parieto-occipital regions of the cortex. This allows us to conclude that the relationship between 5-HTTLPR polymorphism and the ability for behavioral control is mediated by the electrophysiological activity of the corresponding parts of the cortex. It can also be noted that the effect of anxiety was detected only for the left and right, but not the medial part of the parieto-occipital cortex, while the 5-HTTLPR effect was reliably revealed for six selected parts of the cortex, including the medial parieto-occipital and all frontal regions. On this basis, it can be argued that although both the anxiety effect and the allelic polymorphism of the serotonin transporter are equally manifested in the amplitude of the premotor potential, they have different topography in the cortical areas – the anxiety effect affects a significantly narrower area of the cortex than the allelic polymorphism effect. As for the effects of gender and ethnic-regional affiliation of subjects on the amplitude of brain responses, we have so far failed to separate them from the effect of allelic polymorphism, or to describe the mechanism of their interaction. All three factors affect the amplitude of the premotor potential in the same areas of the cortex and at the same time intervals of the brain reaction. Therefore, at the present stage of the study, we can only conclude that the statistical model we have chosen for the pairwise assessment of the effects of various factors on the neurophysiological processes that underlie the voluntary control of movements in the stop-signal paradigm did not allow us to achieve the study aim and identify the brain mechanism of their interaction.

## Conclusion

Allelic polymorphism 5-HTTLPR is associated simultaneously with an increased level of personal anxiety and with a better ability to control movements in experimental conditions associated with the need to make decisions with a lack of time. It can be hypothesized that the S allele of the serotonin transporter is associated with better adaptability to living conditions under conditions of increased danger, which is indirectly confirmed by the frequency of occurrence of this allele in various ethno-regional groups. Analysis of neurophysiological processes recorded by EEG assessment recorded under the stop-signal paradigm showed that both the level of anxiety and 5-HTTLPR polymorphism affect the amplitude of the premotor readiness, but the topography of the effects of anxiety and polymorphism is significantly different.

## Conflict of interest

The authors declare no conflict of interest.
